# The engagement of CTLA-4 on primary melanoma cell lines induces antibody-dependent cellular cytotoxicity and TNF-α production

**DOI:** 10.1186/1479-5876-11-108

**Published:** 2013-05-01

**Authors:** Stefania Laurent, Paola Queirolo, Silvia Boero, Sandra Salvi, Patrizia Piccioli, Simona Boccardo, Simona Minghelli, Anna Morabito, Vincenzo Fontana, Gabriella Pietra, Paolo Carrega, Nicoletta Ferrari, Francesca Tosetti, Lung-Ji Chang, Maria Cristina Mingari, Guido Ferlazzo, Alessandro Poggi, Maria Pia Pistillo

**Affiliations:** 1 Department of Internal Medicine (DIMI), University of Genoa, Genoa, Italy; 2 Department of Medical Oncology A, IRCCS AOU San Martino-IST, Genoa, Italy; 3 Unit of Molecular Oncology and Angiogenesis, IRCCS AOU San Martino-IST, Genoa, Italy; 4 Department of Pathology, IRCCS AOU San Martino-IST, Genoa, Italy; 5 G. Gaslini Institute, Genoa, Italy; 6 Unit of Tumor Genetics and Epigenetics, IRCCS AOU San Martino-IST, Genoa, Italy; 7 Unit of Epidemiology and Biostatistics, IRCCS AOU San Martino-IST, Genoa, Italy; 8 Department of Experimental Medicine, University of Genoa, Genoa, Italy; 9 Department of Molecular Genetics and Microbiology, University of Florida, Gainesville, FL, USA; 10 Unit of Immunology, IRCCS AOU San Martino-IST, Genoa, Italy; 11 Department of Human Pathology, School of Medicine, University of Messina, Messina, Italy

**Keywords:** CTLA-4, Melanoma, Ipilimumab, ADCC, NK/γδ T cell activation

## Abstract

**Background:**

CTLA-4 (Cytotoxic T lymphocyte antigen-4) is traditionally known as a negative regulator of T cell activation. The blocking of CTLA-4 using human monoclonal antibodies, such as Ipilimumab, is currently used to relieve CTLA-4-mediated inhibition of anti-tumor immune response in metastatic melanoma. Herein, we have analyzed CTLA-4 expression and Ipilimumab reactivity on melanoma cell lines and tumor tissues from cutaneous melanoma patients. Then, we investigated whether Ipilimumab can trigger innate immunity in terms of antibody dependent cellular cytotoxicity (ADCC) or Tumor Necrosis Factor (TNF)-α release. Finally, a xenograft murine model was set up to determine *in vivo* the effects of Ipilimumab and NK cells on melanoma.

**Methods:**

CTLA-4 expression and Ipilimumab reactivity were analyzed on 17 melanoma cell lines (14 primary and 3 long-term cell lines) by cytofluorimetry and on 33 melanoma tissues by immunohistochemistry. CTLA-4 transcripts were analyzed by quantitative RT-PCR. Soluble CTLA-4 and TNF-α were tested by ELISA. Peripheral blood mononuclear cells (PBMC), NK and γδT cells were tested in ADCC assay with Ipilimumab and melanoma cell lines*.* TNF-α release was analyzed in NK-melanoma cell co-cultures in the presence of ipilimumab. *In vivo* experiments of xenotransplantation were carried out in NOD/SCID mice. Results were analyzed using unpaired Student’s t-test.

**Results:**

All melanoma cell lines expressed mRNA and cytoplasmic CTLA-4 but surface reactivity with Ipilimumab was quite heterogeneous. Accordingly, about 2/3 of melanoma specimens expressed CTLA-4 at different level of intensity.

Ipilimumab triggered, via FcγReceptorIIIA (CD16), ex vivo NK cells as well as PBMC, IL-2 activated NK and γδT cells to ADCC of CTLA-4^+^ melanoma cells. No ADCC was detected upon interaction with CTLA-4^-^ FO-1 melanoma cell line. TNF-α was released upon interaction of NK cells with CTLA-4^+^ melanoma cell lines. Remarkably, Ipilimumab neither affected proliferation and viability nor triggered ADCC of CTLA-4^+^ T lymphocytes. In a chimeric murine xenograft model, the co-engraftment of Ipilimumab-treated melanoma cells with human allogeneic NK cells delayed and significantly reduced tumor growth, as compared to mice receiving control xenografts.

**Conclusions:**

Our studies demonstrate that Ipilimumab triggers effector lymphocytes to cytotoxicity and TNF-α release. These findings suggest that Ipilimumab, besides blocking CTLA-4, can directly activate the elimination of CTLA-4^+^ melanomas.

## Background

Cytotoxic T lymphocyte antigen-4 (CTLA-4) is a glycoprotein of the immunoglobulin superfamily regarded as the main inhibitory receptor of T cell activation and effector function. CTLA-4 is expressed on the surface of T cells upon activation and its engagement with B7 ligands (CD80/CD86), expressed on antigen presenting cells (APC), inhibits cell proliferation, cytokine production and cell cycle progression [1,2]. Several mechanisms could explain the ability of CTLA**-**4 to inhibit T cell function ranging from prevention of CD28-mediated positive T cell co-stimulation, interference with TCR function or interaction with signaling molecules [3].

CTLA-4 is also expressed on a subset of T cells with immunosuppressive properties (regulatory T cells; Tregs) [4] and on different types of non-T cells, both normal [5-8] and neoplastic [9-14]. We had previously reported CTLA-4 constitutive expression on established cell lines derived from different solid tumors, including melanoma. We also showed that CTLA-4 engagement with B7 ligands induces tumor cell death through apoptosis [11] suggesting a functional role of CTLA-4 molecule also in tumor cells.

The blocking of the physiological inhibitory function of CTLA-4 in T cells is the rationale for the employment of antagonistic anti-CTLA-4 mAbs as therapeutic tools to treat different solid tumors [15], mainly metastatic melanoma [16,17]. Indeed, this approach is supported by preclinical studies showing induction of durable antitumor T cell immunity following treatment with anti-CTLA-4 mAbs [18,19]. By blocking the interaction between CTLA-4 expressed by T cells and B7 ligands expressed by APC, these mAbs may promote further activation and expansion of tumor-specific T cells [20,21]. In particular, CTLA-4/B7 blocking in murine models results in increased IL-2 and interferon-gamma (IFN-γ) production by lymphocytes, increased expression of major histocompatibility complex (MHC) class I molecules, and markedly increased tumor killing [22,23]. The CTLA-4 blockade may also prevent the reverse negative signaling provided by the interaction of CTLA-4 expressed on Tregs with B7 expressed on dendritic cells [24,25] or CD4^+^ T cells [26].

Two human anti-CTLA-4 IgG mAbs, Ipilimumab (Bristol-Myers Squibb, Princeton, NJ) and Tremelimumab (Pfizer, New York, NY), have been used, either alone or in combination with vaccines, in the immunotherapy of melanoma [16,17]. Ipilimumab, approved by the US Food and Drug Administration for the treatment of metastatic melanoma [27], has been the anti-CTLA-4 mAb most extensively investigated, although the molecular mechanisms underlying its anti-tumor activity have not been fully elucidated.

It has been suggested that both Ipilimumab and Tremelimumab inhibit CTLA-4 negative signaling without inducing a cytotoxic effect on T cells [28,29]. These reports are mainly based on the fact that CTLA-4 blockade does not seem to reduce the absolute number of total CD4^+^ T cells and/or to deplete the Treg repertoire in the *in vivo* studies [28,30]. Nevertheless, whether human anti-CTLA-4 antibodies could induce ADCC of CTLA-4^+^ melanoma cell targets has not yet been investigated.

Herein, we show that patient-derived melanoma cells and tissues constitutively express CTLA-4 molecule. We demonstrate that CTLA-4 engagement with Ipilimumab triggers innate immune cells to ADCC of CTLA-4^+^ melanoma cells and Tumor Necrosis Factor (TNF)-α production. That NK cells may be involved in the elimination of CTLA-4^+^ melanoma cells it has been confirmed in a chimeric murine xenograft model as well.

## Methods

### Primary and established cell lines

Primary melanoma cell lines were derived from tumor tissue samples of cutaneous melanoma patients, who underwent surgical resection of skin or lymph node metastases at the IRCCS AOU San Martino-IST (Genoa, Italy). This study was approved by the local Institutional Ethics Committee (n.OMA09.001) and patients gave written informed consent according to the Declaration of Helsinki.

Tissue specimens were processed for establishment of the primary cell lines as described [31].

Expression of Melan-A and GP100 melanocyte differentiation antigens (MDA), of CD133, CD117 and CD271 stem cell-related antigens (SCA), of nestin and CD56 neural crest antigens (NCA) was analyzed by immunofluorescence, as reported [32] and described in Additional file 1.

Among the established melanoma cell lines, C32 and MeWo were obtained from ECACC (Salisbury, UK) and FO-1 was kindly provided by S. Ferrone (New York Medical College, 1991), HLA typed by SSPO analysis [33] and authenticated in our lab by PCR-SSP. The human lymphoblastoid B cell line C1R-neo was obtained from ATCC (Manassas, USA, 2011) and validated according to its short tandem repeat. Last authentication was performed before using the cell lines for the present study.

### Analysis of CTLA-4 expression by flow cytometry

Expression of surface and cytoplasmic CTLA-4 was analyzed by flow cytometry as reported [8] and described in Additional file 1. For CTLA-4 surface staining with Ipilimumab human antibody (Bristol-Myers-Squibb), indirect immunofluorescence was performed by incubating, for 30 min at 4°C, 2×10^5^ cells/sample with the mAb (20 μg/ml). CTLA-4 cytoplasmic staining with Ipilimumab was performed on fixed (2% paraformaldehyde) and permeabilized (0.1% saponin) 4×10^5^ cells/sample. Both stainings were followed by the addition of Alexafluor 647-conjugated goat anti-human IgG secondary antibody (Molecular Probes, Inc. Eugene, OR, USA). Negative controls included directly labelled and unlabeled isotype-matched irrelevant mAbs.

Results were expressed as mean ratio of relative fluorescence intensity (MRFI), calculated as follows: mean fluorescence intensity (MFI) of CTLA-4 staining/MFI of irrelevant isotype-matched mAb staining.

### Analysis of CTLA-4 transcripts by RT-PCR and qRT- PCR

Analysis of CTLA-4 transcript variants by RT-PCR and quantitative RT-PCR (qRT-PCR) were performed as described in Additional file 1 and in the Table of Additional file 2.

### Analysis of CTLA-4 expression by immunohistochemistry

Immunohistochemical (IHC) analysis of CTLA-4 expression was performed on formalin-fixed, paraffin-embedded (FFPE) tissues of cutaneous melanoma lesions by staining with either the anti-CTLA-4 14D3 mAb or Ipilimumab.

For reaction development, we used an Alkaline Phosphatase(AP)**-**Fast Red staining for 14D3 and a peroxidase-DAB staining for Ipilimumab. Both whole tissue slides and tissue microarray (TMA) were stained (see Additional file 1). Scores for percentage of stained cells were 0 (negative), 1 (1-29%), 2 (30-59%), 3 (60-100%). Scores for staining intensity were 0 (negative), 1+ (weak), 2+ (moderate) 3+ (strong). A final immunoreactive score (IRS) for CTLA-4 expression was obtained by multiplying both scores [34] resulting in the following IRS (values from 0 to 9): 0 (negative), 1–4 (low to intermediate) and ≥6 (high). Stained slides were analyzed by two independent observers under an optical microscope (Olympus BX41) using 10× ocular lens, 63× objectives. Image acquisition was performed with Leica (DMD1.08) microscope.

### Analysis of soluble CTLA-4 by ELISA

Soluble CTLA-4 (sCTLA-4) secreted by the melanoma cells was measured in culture supernatants (SN) by using a sCTLA-4-specific ELISA kit (Bender MedSystems**,** Milan, Italy) according to manufacturer’s protocol. SN were collected from melanoma cells, grown to approximately 80% confluence, and tested undiluted in duplicate. The lowest sensitivity threshold of the assay was 0.13 ng/ml.

### Leukocyte cell separation, antibody-dependent cellular cytotoxicity (ADCC) and TNF-α production assays

Peripheral blood mononuclear cells (PBMC) were obtained after Ficoll-Hypaque density centrifugation of blood samples derived from healthy volunteers. Highly purified preparations of NK cells and γδT cells were obtained from PBMC as described [35] and tested in a conventional 4h ADCC assay [36]. Production of TNF-α was determined by ELISA (see Additional file 1).

### Chimeric xenograft NOD/SCID model

Non-obese diabetic/severe combined immunodeficiency (NOD**/**SCID) mice were purchased from Harlan Laboratories (Udine, Italy) and housed according to the institutional animal care guidelines. All experiments were approved by the Ethics Committee for Animal Use in Cancer Research at our institute. All mice were approximately 7 weeks-old. Tumorigenicity assay of melanoma cell lines was performed as described in Additional file 1.

Different melanoma xenografts were prepared for subcutaneous (s.c.) injections into NOD/SCID mice. Briefly, freshly harvested MECO cells (2×10^6^) were washed twice and incubated with Ipilimumab or Rituximab (both at 20 μg/ml) at 4°C for 30 min. Treated and untreated MECO cells, either alone or mixed at 1:1 ratio with human NK cells isolated from the buffy coats of three different healthy donors, were injected s.c. (200 μl/mouse) into the mice (6 injections per each experimental condition). Tumor growth was evaluated as described in Additional file 1 starting from day 5 of melanoma and NK cell xenograft implantation.

### Statistical analyses

Results were analyzed using unpaired Student’s t-test. Pairwise correlation was assessed through Spearman's nonparametric coefficient. All tests were two-tailed and data were analyzed using the Stata software. Statistical significance was accepted for any *P value* < 0.05.

## Results

### CTLA-4 is expressed by primary cutaneous melanoma cell lines

In the present study, we analyzed CTLA-4 expression in 14 primary cell lines originating from metastatic lesions of cutaneous melanoma patients and in 3 long-term established melanoma cell lines.

**Figure 1:**
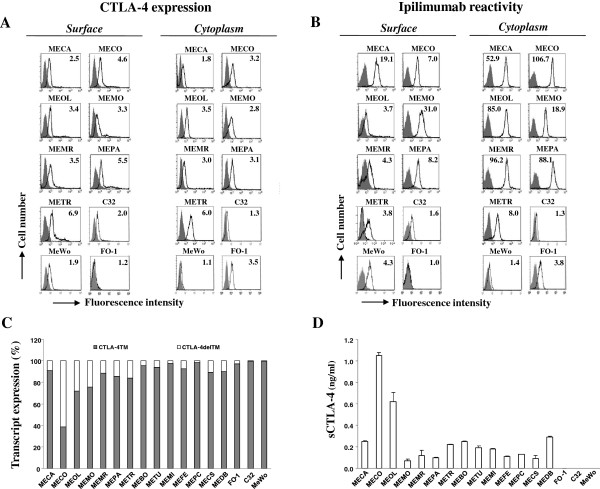
**CTLA-4 is expressed in cutaneous melanoma cell lines and it is recognized by Ipilimumab.** Flow cytometric profiles of representative primary cell lines and established cell lines (C32, MeWo, FO-1), stained with either an anti-CTLA-4 polyclonal antiserum (surface) and 14D3 mAb (cytoplasm) (panel **A**) or Ipilimumab Ab (panel **B**). FO-1 cell line was used as control for CTLA-4 surface negative expression. Open histograms represent staining with specific anti-CTLA-4 Abs, filled histograms represent staining with isotype control Abs. Numbers within the quadrants represent values of MRFI. Panel **C**, densitometric analysis of RT-PCR for CTLA-4 transcripts (TM and delTM) normalized to GAPDH and reported as % of expression. The experiment was repeated three times with similar results. Panel **D**, ELISA analysis of sCTLA-4 in melanoma cell SN. Results are means ± SD of two independent experiments performed in duplicate.

We found that all the cell lines, except FO-1, expressed variable levels of surface and cytoplasmic CTLA-4 (Figure 1A). It is of note that FO-1 cell line appeared to be surface CTLA-4 negative but it was positive in the cytoplasm (Figure 1A).

We next investigated whether CTLA-4 expressed by melanoma cells was recognized by the therapeutic Ipilimumab antibody. Flow cytometric analysis showed that Ipilimumab reacted, with different intensity, at the cell surface of all, except FO-1, melanoma cell lines tested (Figure 1B). It is of note that Ipilimumab did react with FO-1 into the cytoplasm. Both polyclonal and Ipilimumab anti-CTLA-4 antibodies reacted with the established human melanoma cell lines C32 and MeWo (Figure 1A,B).

**Figure 2:**
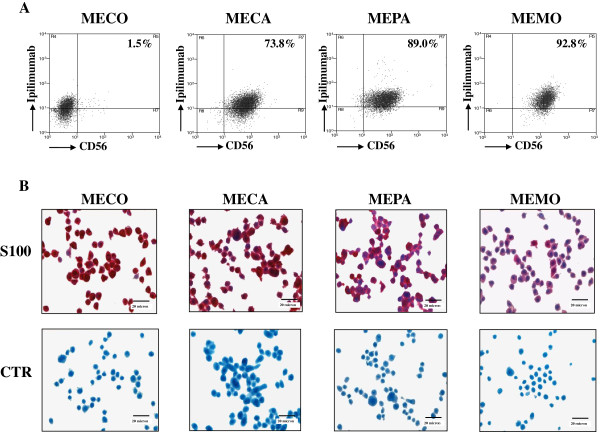
**Analysis of CD56 and S100 expression in CTLA-4 positive melanoma cell lines.** Panel **A**, cytofluorimetric analysis of direct and indirect immunofluorescent co-stainings performed with a PE-conjugated anti-CD56 (NCAM) and Ipilimumab Abs, as shown for representative cell lines. Numbers within the quadrants represent the percentage of Ipilimumab/CD56 double positive melanoma cells. Panel **B**, immunohistochemical staining with anti-S100 mAb and with anti-CD20 mAb used as isotype-matched irrelevant antibody providing the negative control (CTR), as shown for representative cell lines. Images were taken using magnification 400×. Scale bar, 20 micron.

CTLA-4 expression in primary melanoma cell lines appeared to be independent from the stage of melanoma differentiation. Indeed, cytofluorimetric analysis did not point out any statistically significant Spearman’s correlation coefficient (not shown) for surface CTLA-4 expression, detected with either the polyclonal antiserum or Ipilimumab, and the expression pattern of MDA, as well as of SCA and NCA (Table 1). Flow cytometric analysis of melanoma cells, double-stained with Ipilimumab and an anti-CD56 (NCAM) Ab, showed that the cell lines expressed CTLA-4 and CD56 simultaneously (representative profiles are shown in Figure 2A), although at variable intensity (Table 1). The melanoma nature of our cell lines was further confirmed by IHC staining for S100 marker (representative experiments are shown in Figure 2B).

**Table 1:** Expression patterns of CTLA-4, MDA, SCA and NCA in 14 primary melanoma cell lines

**Melanoma cell line**	**CTLA-4**		**MDA**		**SCA**			**NCA**	
	***Polyclonal antiserum***	***Ipilimumab***	***Melan A***	***GP100***	***CD133***	***CD117***	***CD271***	***Nestin***	***CD56***
**MECA**	2.5	19.1	4.2	7.5	0.9	0.7	2.7	4.6	18.8
**MECO**	4.6	7.0	25.7	17.0	1.0	0.7	3.2	4.0	1.5
**MEOL**	3.4	3.7	10.9	2.5	1.1	0.6	26.8	6.1	24.5
**MEMO**	3.3	31.0	1.1	2.2	1.1	0.8	90.0	11.4	42.5
**MEMR**	3.5	4.3	2.4	2.1	0.9	0.9	1.0	1.9	32.0
**MEPA**	5.5	8.2	2.5	1.8	1.0	0.8	13.9	7.4	14.7
**METR**	6.9	3.8	18.8	23.6	10.3	0.8	12.5	2.3	1.4
**MEBO**	1.9	6.7	1.2	4.2	1.7	17.5	1.0	14.5	3.7
**METU**	3.5	7.0	1.0	1.0	1.3	2.0	22.5	9.5	12.0
**MEMI**	1.7	2.9	19.9	2.2	1.4	70.2	1.0	62.2	20.4
**MEFE**	2.0	7.2	22.8	1.0	1.0	1.6	18.3	2.2	46.9
**MEPC**	2.4	1.4	11.8	1.0	1.6	0.7	72.9	1.4	1.6
**MECS**	5.8	2.9	6.8	1.2	1.0	6.3	19.9	1.2	12.3
**MEDB**	3.7	1.7	0.8	2.7	1.0	8.5	4.8	1.9	2.6

The 14 primary melanoma cell lines derived from metastatic lesions were stained with the indicated anti-CTLA-4 antibodies and analyzed by flow cytometry for the expression patterns of melanoma differentiation antigens (MDA), stem cell-related antigens (SCA) and neural crest antigens (NCA).

Results were expressed as mean ratio of relative fluorescence intensity (MRFI), calculated as follows: mean fluorescence intensity (MFI) of CTLA-4 staining/MFI of irrelevant isotype-matched mAb staining.

CTLA-4 expression was confirmed at transcriptional level by RT-PCR analysis identifying the two transcript variants CTLA-4TM and CTLA-4delTM/soluble [37].

Expression of CTLA-4TM transcript was found with variable intensity in all melanoma cell lines; CTLA-4delTM (sCTLA-4) transcript was expressed at lower levels in respect to CTLA-4TM, in all cell lines except in MECO (Figure 1C). The expression of CTLA-4delTM transcript confirmed the finding that the primary melanoma cell lines secreted detectable levels of sCTLA-4 (range: 0.1-1.05 ng/ml), as defined by ELISA (Figure 1D).

### CTLA-4 is expressed by cutaneous melanoma tissues

**Figure 3:**
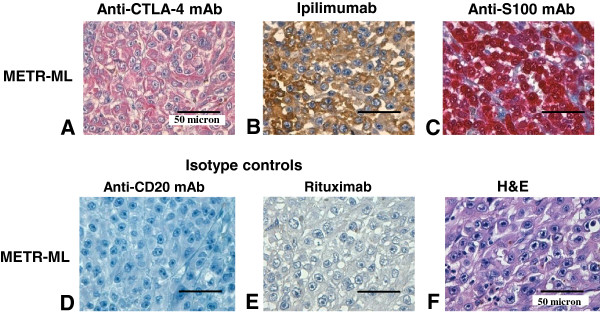
**CTLA-4 is expressed in cutaneous melanoma tissues and it is recognized by Ipilimumab.** Immunohistochemical (IHC) analysis is shown in a representative metastatic melanoma lesion (ML). IHC staining with murine anti-CTLA-4 14D3 mAb of METR-ML (**A**) was detected with AP-conjugated secondary antibody and Fast Red chromogen (red staining). IHC staining with Ipilimumab of METR-ML (**B**) was detected with HRP-conjugated secondary antibody and DAB chromogen (brown staining). IHC staining with anti-S100 mAb was used as positive control as shown in the representative METR-ML (**C**) sample. IHC stainings with a murine anti-CD20 mAb and human Rituximab anti-CD20 mAb were used as isotype negative controls for 14D3 and Ipilimumab, respectively, as shown for METR-ML (**D,E**). Images were taken using magnification 630×. Scale bar, 50 micron.

CTLA-4 protein expression was also found on FFPE tissue sections from 5 metastatic lesions (ML) used to originate the cell lines, and from additional melanoma lesions. IHC with a murine anti-CTLA-4 mAb (14D3) demonstrated a diffuse and strong positivity, uniformly spread throughout the tumor (representative staining in Figure 3A). A similar staining pattern was observed with Ipilimumab (representative staining in Figure 3B).

An anti-S100 mAb was used as positive control (representative staining in Figure 3C) whereas a murine anti-CD20 mAb and Rituximab were used as isotype-matched irrelevant mAbs (representative staining in Figure 3D,E).

Moreover, we analyzed 28 melanoma tissues by TMA immunostaining for CTLA-4 and we evaluated CTLA-4 expression through the immunoreactive score (IRS; 0 = negative, 1-4 = low to intermediate and ≥6 = high), an index which takes into account both parameters of percentage of positively stained cells and staining intensity, according to their individual scores (34), as described in the Methods. Twenty out of 28 (71.4%) tissues were found positive for CTLA-4 expression (IRS > 1), although with variable percentage of stained cells and intensity, whereas 8 (28.6%) were found CTLA-4-negative (IRS = 0) (Table 2). In particular, 9 out of 20 (45.0%) CTLA-4-positive tissues showed low to intermediate expression (IRS 1–3) and 11 (55.0%) showed high CTLA-4 expression (IRS ≥6).

**Table 2:** CTLA-4 expression in melanoma tissue microarray by immunohistochemistry

**Melanoma cases**	**CTLA-4 staining**		**IRS**
	***% of positive cells***	***Intensity***	
1	10	3+	3
2	70	2+	6
3	10	1+	1
4	30	1+	2
5	5	1+	1
6	80	2+	6
7	70	2+	6
8	70	2+	6
9	80	2+	6
10	0	0	0
11	85	3+	9
12	0	0	0
13	10	1+	1
14	0	0	0
15	0	0	0
16	0	0	0
17	5	1+	1
18	5	1+	1
19	0	0	0
20	60	2+	6
21	90	2+	6
22	70	2+	6
23	30	1+	2
24	0	0	0
25	100	1+	1
26	0	0	0
27	80	3+	9
28	95	3+	9

CTLA-4 expression by IHC staining of tissue microarrays consisting of 28 formalin-fixed, paraffin-embedded melanoma sections.

IHC staining was performed with the murine anti-CTLA-4 14D3 mAb and detected with AP-conjugated secondary antibody and Fast Red chromogen.

IRS: immunoreactive score (values from 0 to 9) for CTLA-4 expression was as follows: 0 (negative), 1–4 (low to intermediate), ≥6 (high). IRS score was obtained by multiplying the scores of % of positively stained cells and staining intensity. Scores for % of stained cells were: 0 (negative), 1 (1-29%), 2 (30-59%), 3 (60-100%). Scores for staining intensity were: 0 (negative), 1+ (weak), 2+ (moderate), 3+ (strong).

We further confirmed the expression of CTLA-4 and reactivity of Ipilimumab by performing qRT-PCR in a CTLA-4^+^ melanoma tissue sample consisting of almost, if not all, melanoma cells. A strong expression of CTLA-4 transcript was detected in this tissue as compared to METR cell line and FO-1 used as control reference (see Figure of Additional file 3 and Additional file 4).

### Ipilimumab triggers lysis of melanoma cells through ADCC of ex-vivo isolated NK cells

**Figure 4:**
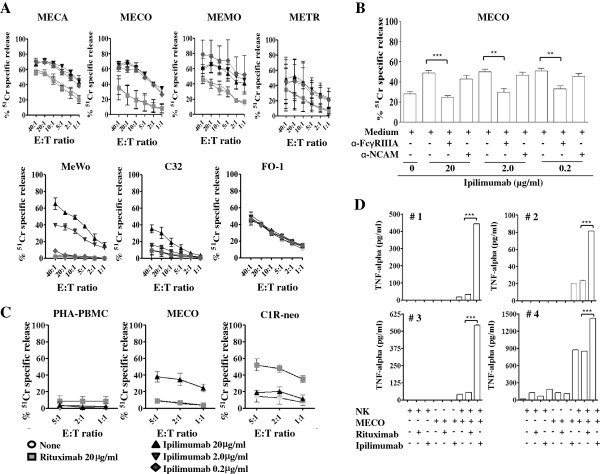
**Ipilimumab triggers ex-vivo isolated NK cells to kill melanoma cells and to secrete TNF-α.** Panel **A**, NK cells were analyzed for their cytolytic activity in an ADCC assay using Ipilimumab (0.2-2.0-20 μg/ml) and melanoma primary cell lines (MECA, MECO, MEMO, METR), as well as established cell lines (MeWo, C32 and FO-1) at different effector to target cell ratio (E:T = 40:1, 20:1, 10:1, 5:1, 2:1, 1:1). NK cells were incubated with target cells in medium alone (none) or in the presence of Rituximab used as isotype-matched control Ab. Results are expressed as % of ^51^Cr specific release and are the mean ± SD of experiments with 6 donors. Panel **B**, effect of the addition of anti-FcγRIIIA mAb (α-FcγRIIIA) on the ADCC triggered with Ipilimumab (at the indicated doses) using ex-vivo NK cells and CTLA-4^+^ MECO target cells. Results obtained by adding an anti-NCAM mAb (α-NCAM), as isotype-matched control Ab for the anti-FcγRIIIA mAb, to ADCC assay are shown. ***P < 0.0001, **P < 0.0002. Panel **C**, lysis of PHA-stimulated PBMC or MECO or C1R-neo CD20^+^ B cells using ex-vivo NK cells alone (none) or with Ipilimumab or Rituximab (at 20 μg/ml). Panel **D**, NK cells, ex-vivo isolated from 4 different healthy donors, were incubated with MECO at the E:T ratio of 1:1 alone or in the presence of Ipilimumab or Rituximab (2.0 μg/ml) for 24 h. Then, culture SN were harvested and analyzed by ELISA for the presence of TNF-α. Results are expressed as pg/ml of TNF-α/10^5^ NK cells. Statistical significance: ***P < 0.001, **P < 0.005.

We further investigated whether Ipilimumab could trigger activation of NK cells to ADCC upon interaction with CTLA-4^+^ melanoma cells. To this aim, ex-vivo isolated NK cells were used in a conventional cytolytic assay using CTLA-4^+^ melanoma cell lines in the presence of Ipilimumab. The results showed that NK cells efficiently killed melanoma cells at high effector:target cell ratio (E:T) (40-60% of lysis at 40:1 E:T ratio) (Figure 4A). This lysis was barely detectable at very low E:T ratio of 1:1 (5-17% depending on cell line used), but it was significantly increased by the addition of Ipilimumab to the cytolytic assay. Indeed, the lysis of MECA, MECO, MEMO and METR cell lines at 1:1 E:T ratio (21, 7, 15 and 5% respectively in the absence of antibody) was enhanced in the presence of Ipilimumab (46, 35, 42 and 23% respectively) (Ipilimumab *vs.* control, *P* < 0.001; *P* = 0.023; *P* = 0.003; *P* = 0.023, respectively, referred to 2.0 μg/ml of Ipilimumab) (Figure 4A). Moreover, it appeared that this enhancement was superimposable at concentration of 20, 2.0 and 0.2 μg/ml of Ipilimumab.

The induction with Ipilimumab of NK cell-mediated ADCC was confirmed using the established melanoma cell lines C32 and MeWo (Figure 4A). It is of note that Ipilimumab did not trigger ADCC of the CTLA-4 negative cell line FO-1 (Figure 4A). Indeed, NK cell-mediated lysis of FO-1, in the presence of Ipilimumab, was superimposable to basal cell lysis observed in the absence of any antibody.

The triggering of ADCC was detectable only using Ipilimumab as the addition of the human anti-CD20 Rituximab antibody to the cytolytic assay did not affect basal lysis of the different melanoma cell lines tested (Figure 4A).

In addition, no lysis of melanoma cells was detected in the presence of Ipilumumab alone, avoiding a direct effect of Ipilimumab after a short time incubation (data not shown).

The triggering of ADCC detected using Ipilimumab with ex-vivo NK cells and CTLA-4^+^ melanoma targets was conceivably due to the binding of Ipilimumab to FcγRIIIA expressed on NK cells as the addition of an anti-FcγRIIIA antibody to the assay could almost block the ADCC (Figure 4B*, P <* 0.0001). The addition of an anti-NCAM antibody did not affect Ipilimumab-mediated ADCC (Figure 4B, *P* = 0.129 n.s.). Although not shown, Ipilimumab-triggered ADCC in the presence of human serum was superimposable to that observed in its absence. This indicates that human immunoglobulins do not compete with Ipilimumab bound to CTLA-4 expressed on melanoma cells for the binding with FcγRIIIA.

It is known that CTLA-4 is also expressed on activated T cells and this expression is maximal on day2 after stimulation with PHA [9,38]. Thus, to analyze whether NK cells can kill T cells expressing CTLA-4 as well, we used ex-vivo isolated NK cells as effector cells and PHA-stimulated PBMC as target cells. No lysis of PBMC (Figure 4C, left panel) in the presence of Ipilimumab was detected although CTLA-4 was expressed on these cells (data not shown). Of note, no differences were observed using autologous or allogeneic NK cells. Rituximab used as control did not trigger lysis of PBMC as expected (being CD20 not expressed on PHA blasts), while it efficiently triggered lysis of CD20^+^ C1R-neo lymphoblastoid cell line (Figure 4C, right panel).

### Ipilimumab triggers NK cells to produce TNF-α upon interaction with melanoma cells

We next analyzed whether Ipilimumab could also trigger ex-vivo NK cells to produce anti-tumor cytokines, such as TNF-α, during co-cultures with CTLA-4^+^ melanoma cells. To this aim, TNF-α was measured in the SN obtained from co-cultures of NK cells isolated from 4 different donors and CTLA-4^+^ MECO cell line (1:1 E:T ratio), by ELISA (Figure 4D). The addition of Ipilimumab to NK-MECO cell co-cultures strongly enhanced TNF-α production as compared to NK-MECO co-cultures alone or in the presence of Rituximab used as control (Ipilimumab *vs.* Rituximab, *P* = 0.041).

### Ipilimumab triggers ex-vivo isolated PBMC, NK or γδT cells cultured with IL-2 to kill melanoma cells through ADCC

**Figure 5:**
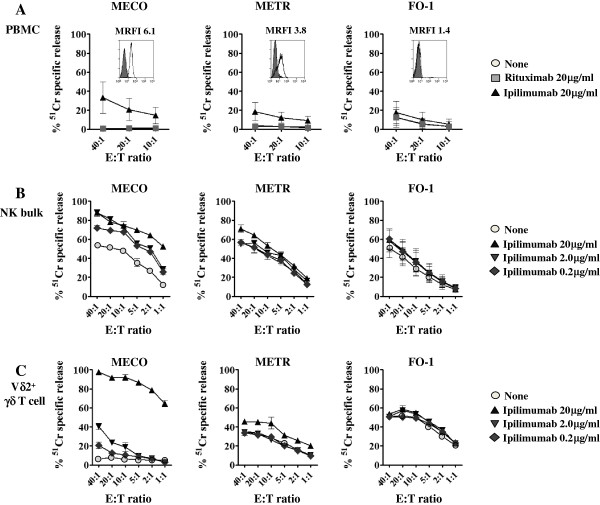
**Ipilimumab-triggered ADCC is dependent on the expression of CTLA-4 on melanoma cells.** PBMC (A, n = 6), IL-2-activated NK (B, n = 4) and Vδ2^+^T cell populations (C, n = 3) were analyzed for ADCC activity using the melanoma cell lines: MECO (left), METR (middle) or FO-1 (right). Experiments were performed at the indicated E:T ratios without (none) or with anti-CTLA-4 Ipilimumab Ab or anti-CD20 Rituximab Ab. Results are expressed as % of ^51^Cr specific release and are the mean ± SD of results obtained with different effectors. Inserts in the first row of histograms show the reactivity of Ipilimumab (MRFI) with the indicated cell lines. Reactivity was analyzed by indirect immunofluorescence on a cytofluorimeter. Open histograms or filled histograms represent staining with either anti-CTLA-4 Ipilimumab Ab or isotype control Ab respectively.

We next analyzed whether ADCC can also be elicited in PBMC, IL-2 activated NK cells and γδT cell populations expressing FcγRIIIA. Furthermore, we analyzed ADCC using three different melanoma cell lines expressing different levels of reactivity with Ipilimumab. Indeed, as shown in Figure 5A, MECO cell line expressed high reactivity with Ipilimumab (Figure 5A, MRFI:6.1) while FO-1 melanoma cells did not react with Ipilimumab (Figure 5A, MRFI:1.4) and METR cells showed an intermediate reactivity with Ipilimumab (Figure 5A, MRFI:3.8).

PBMC can efficiently kill MECO at E:T ratio of 40:1 in the presence of Ipilimumab (35% *vs.* 1% of lysis in the absence of antibody, *P <* 0.001). It is of note that Ipilimumab-mediated ADCC was still evident at 20:1 and detectable at 10:1 (20% and 12% *vs.* 1% of lysis, respectively, in the absence of antibody, *P =* 0.002 and *P =* 0.003).

No difference between basal lysis and lysis in the presence of Ipilimumab was detectable using the CTLA-4 surface negative FO-1 cell line. Using METR as target cells, we detected, at the E:T target ratio of 40:1, an increment of lysis in the presence of Ipilimumab (from 5% to 20%, *P =* 0.004). This increase was lower at 20:1 (from 5% to 11%, *P =* 0.003) and almost undetectable at 10:1 (from 5% to 8%, *P =* 0.006) E:T ratios respectively. IL-2 activated NK cells (Figure 5B) killed more efficiently MECO than METR in the presence of Ipilimumab (at 5:1 E:T ratio from the basal lysis of 35% to 75% with Ipilimumab using MECO and from the basal lysis of 35% to 42% with Ipilimumab using METR as target, *P <* 0.001 and *P =* 0.228 n.s., respectively). No increment of lysis was detected with Ipilimumab using the CTLA-4 surface negative FO-1 cells. Finally, γδT cell populations expressing FcγRIIIA can kill very efficiently the MECO cell line in the presence of Ipilimumab (from 5% as basal level to 65% with Ipilimumab at 1:1 E:T ratio, *P <* 0.001). On the other hand, the lysis of METR was barely incremented in the presence of Ipilimumab while no effect was detected on the lysis of FO-1 (Figure 5C, middle and right panel). In no instance, Rituximab used as control antibody, could significantly enhance cytolysis of the melanoma cell lines using different effector cells (Figure 5A and not shown).

### Ex-vivo NK cells in the presence of Ipilimumab reduces melanoma cell growth in a melanoma/NK cell xenograft model

We next investigated *in vivo*, using a NOD/SCID murine xenograft model, whether NK cells can affect tumor growth in the presence of Ipilimumab. *In vivo* experiments were carried out with the MECO cells as this cell line efficiently triggered *in vitro* NK cell-mediated ADCC (Figure 4A).

Previous studies showed that human NK cells engraft and retain cytotoxic function when injected s.c. into SCID mice, along with allogeneic human tumor cells [39]. Thus, we injected s.c. a mixed cell suspension of ex-vivo isolated allogeneic human NK cells and MECO cell line incubated in medium alone (MECO/NK) or with Ipilimumab (IPI-MECO/NK) or Rituximab (used as control antibody) (RIT-MECO/NK) at 1:1 NK:MECO ratio. We choose this experimental setting as it more closely reflected the conditions used in the in vitro cytotoxicity assay (Figure 4A). Also, MECO cell line incubated in medium or with Ipilimumab (IPI-MECO) or Rituximab (RIT-MECO) was injected as additional control.

**Figure 6:**
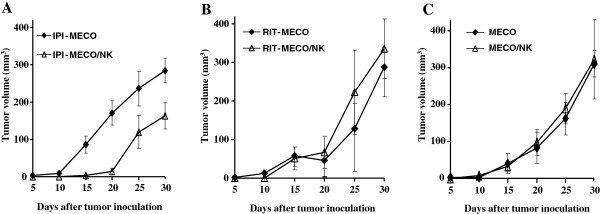
**Ipilimumab triggers NK cells to kill melanoma cells in a chimeric murine xenograft model.** NOD/SCID mice were s.c. injected with melanoma MECO cells (2×10^6^) after incubation with Ipilimumab (IPI-MECO xenograft) or Rituximab (RIT-MECO xenograft) with human allogeneic NK cells (IPI-MECO/NK and RIT-MECO/NK xenografts). Human NK cells were ex vivo isolated from the peripheral blood of 3 different healthy donors and mixed at 1:1 NK:MECO ratio. Controls consisting of MECO cells alone or MECO cells mixed with NK cells (MECO and MECO/NK xenografts, respectively) were also injected. Tumor growth was monitored twice a week and plotted as tumor volumes (mean ± S.E.M.) (panels A, B and C). Data are representative of six independent xenografts with NK cells from the 3 donors (mean ± S.E.M.) from one of the two experiments performed.

After a single inoculation of the six different MECO xenografts (IPI-MECO, IPI-MECO/NK, RIT-MECO, RIT-MECO/NK, MECO and MECO/NK), the tumor growth was monitored weekly for up to 30 days. In all the experimental groups, tumors were detected within 15 days (mean tumor volume: 56.93 ± 11.71 mm^3^) except in IPI-MECO/NK xenografts in which just palpable really small tumors started to appear at that time (mean tumor volume 3.00 ± 2.19 mm^3^, *P* = 0.042 *vs.* all other xenografts). In all mice, tumor growth progressively increased until day 30 (mean tumor volume: 307.11 ± 28.58 mm^3^) but, again, a significantly reduced tumor growth was observed in mice injected with IPI-MECO/NK xenografts (mean tumor volume: 163.15 ± 35.22, *P* = 0.024 *vs.* all other xenografts). Comparing the growth of IPI-MECO/NK xenografts to that of IPI-MECO control xenografts, we found a significant reduction of tumor growth at day 15 (*P* = 0.005), day 20 (*P* = 0.009) and day 30 (*P* = 0.028) (Figure 6A). Moreover, NOD/SCID mice engrafted with RIT-MECO/NK, used as control, did not show delay in tumor formation or inhibition of tumor growth compared with mice engrafted with RIT-MECO (*P* = 0.686) (Figure 6B). It is of note that MECO and MECO/NK xenografts gave rise to tumors of similar volume indicating that NK cells *per se* did not affect tumor cell growth (Figure 6C). The growth of IPI-MECO/NK xenografts was also significantly reduced in respect to the growth of MECO control xenograft as well as to the growth of all other MECO xenografts observed at day 30 (*P* = 0.018 and *P* = 0.042, respectively) (Figure 6A,B,C).

The *in vivo* reduced tumor growth observed with IPI-MECO/NK xenograft was found to correlate with the lytic activity observed *in vitro* when the human allogeneic NK cells used for the *in vivo* injections were co-cultured with MECO cells in the presence of Ipilimumab (data not shown).

## Discussion

In this study, we demonstrate that CTLA-4 is constitutively expressed in a large portion of patient-derived cutaneous melanoma cells, as well as tissues, and it is recognized by Ipilimumab. Furthermore, we show that ADCC and TNF-α secretion are triggered in FcγRIIIA^+^ lymphocyte subsets upon Ipilimumab interaction with CTLA-4 on melanoma cells.

Our data show mRNA and cytoplasmic CTLA-4 expression in all primary melanoma cell lines tested, although the surface CTLA-4 expression was quite heterogeneous (MRFI ranging from 1.2 to 6.9), regardless their stage of differentiation and stemness phenotype. Furthermore, we found, by the TMA approach, that about 2/3 of melanoma tissues expressed CTLA-4. In particular, by using the IRS score, it was possible to differentiate between low-intermediate (45.0%) and high (55.0%) CTLA-4-expressing tissues. The heterogeneity of CTLA-4 expression can be considered as an intrinsic biological characteristic of the tumor. At present, it is not known the physiological role of CTLA-4 on melanoma cells. We have previously demonstrated that CTLA-4 engagement with its natural ligands can deliver an apoptotic signal in haematological and solid tumor cells including melanoma cell lines (11). Thus, the heterogeneity observed in melanoma tissue specimens may be dependent on the selection processes induced by the microenvironment on tumor cells. The heterogeneity of level of CTLA-4 expression on melanoma cell lines may derive from the heterogeneity of the parental tumor tissue from which the cell line has been obtained.

On the other hand, we found that all the melanoma cell lines, but not FO-1, expressed CTLA-4. This strongly suggests that the absence of the tumor microenvironment favours the *in vitro* selection of CTLA-4 positive melanoma cells.

Also CTLA-4 transcripts were found expressed in melanoma tissue sections consisting of melanoma cells without detectable tumor infiltrating lymphocytes. This further reinforces the idea that *in vivo* melanoma cells can express CTLA-4.

Ipilimumab triggered *in vitro* ADCC via the engagement of FcγRIIIA in different effector lymphocyte populations i.e. ex-vivo isolated PBMC, highly purified CD3^-^NK cells, IL-2 activated NK cell bulk populations and γδT lymphocytes. This ADCC led to efficient killing of several melanoma cell lines and it appears that the degree of this process was directly related to the level of CTLA-4 surface expression. This would suggest that a threshold level is necessary for triggering ADCC induced by Ipilimumab. The expression *in vivo* of CTLA-4 on melanoma cells would suggest that ADCC could be triggered also upon *in vivo* administration of Ipilimumab. It is to determine what is this threshold and how/whether ADCC can concur to the outcome of melanoma patients treated with Ipilimumab. Along this line, it has been shown that sera of macaques immunized with a melanoma vaccine could trigger a stronger human PBMC-mediated ADCC of melanoma cells when macaques were vaccinated with melanoma cells together with antibody 11D10 (namely Ipilimumab) compared to macaques vaccinated with only melanoma cells [28]. This ADCC was mainly ascribed to the higher levels of anti-melanoma antibodies present in the sera of macaques [28]. However, the possibility that 11D10 antibody could trigger directly human PBMC-mediated ADCC of melanoma cells has not been analyzed in that report. Indeed, the notion that melanoma cells can express CTLA-4 is more recent [11,12].

We show that γδT cells exerted a stronger ADCC than NK cells. This would depend also on the expression of HLA-I antigens on melanoma target cells. Indeed, it is known that NK cell mediated cytolysis is inhibited by the interaction of specific HLA-I receptors belonging to inhibitory receptor superfamily and self-HLA-I. Thus, ADCC mediated by NK cells would be the balance between positive (through FcγRIIIA) and negative (through HLA-I) signals. On the other hand, γδT cells are not necessarily inhibited upon interaction with HLA-I and thus only the positive triggering signal is evoked leading to a stronger ADCC. To support this interpretation of our results, experiments using self NK and γδT lymphocytes together with autologous melanoma cell lines should be performed.

Activated T cells expressing CTLA-4 were not killed by ADCC most likely due to either the transient or weak expression of CTLA-4 on T cells upon activation [3]. This indicates that Ipilimumab would not impair T cell response exerting its direct effect on melanoma cells by triggering activation of cytolytic effector cells. Our data are not in contrast with the commonly accepted notion that Ipilimumab can block the action of CTLA-4 at the cell surface of T cells; this leads to a stronger immune anti-tumor response that according to previous report is the reason why Ipilimumab is working in patients with melanoma. Indeed, we suggest that the activation of ADCC leading to melanoma cell lysis can concur with the triggering of immune response due to relieve of CTLA-4-mediated down-regulation to a better elimination of melanoma cells.

Further, we show that NOD/SCID mice s.c. co-engrafted with Ipilimumab-coated MECO cells and allogeneic human NK cells had delayed tumor onset and significant inhibition of tumor growth as compared with mice engrafted with Ipilimumab-coated MECO cells alone. These findings suggest that, in our experimental conditions, tumor formation and growth were influenced by the presence of NK cells in the xenograft and that Ipilimumab-mediated ADCC triggering may have played a role as Rituximab, used as antibody control, neither showed delay in tumor formation nor reduction of tumor volume. However, we found that all the mice developed a tumor. The inability of ex-vivo isolated human NK cells to completely suppress tumor cell growth despite the presence of Ipilimumab, may be due to a) the low number of NK cells injected (1:1 NK/melanoma cell ratio); b) the lack of cytokines required for an optimal human NK cell activation as IL-2 or IL-15; c) the lack of accessory immune cells that can aid NK cell in eliminating melanoma cells.

In this regard, it has been reported that CD56^+^ NK cells are more efficient in suppressing the growth of a lung cancer xenograft in SCID mice, if they are coinjected with either CD8^+^ T cells or unfractionated peripheral blood lymphocytes which are presumed to be important for the in situ secretion of NK cell stimulating cytokines [39,40].

Whether the *in vivo* NK cell-mediated antitumor effect occurs via ADCC activity or TNF-α secretion needs further investigations. However, collectively our studies pointed out an involvement of the innate immune system in the antitumor effect of Ipilimumab.

## Conclusions

Herein, we show that patient derived melanoma cell lines and tumor tissues can express CTLA-4. Ipilimumab reacts with CTLA-4 on melanoma cell lines and tissues and is able to trigger antibody dependent cellular cytotoxicity (ADCC) engaging FcγRIIIA on lymphocyte subsets such as primary NK cells, IL-2 activated NK and γδT cells. The degree of ADCC is dependent on the expression level of CTLA-4 on melanoma target cells. Furthermore, NK cells in the presence of Ipilimumab interacting with CTLA-4^+^ melanoma cells can release TNF-α.

These findings can have important therapeutic implications as they suggest 1) a new mechanism of action of Ipilimumab; indeed, although formerly regarded as a CTLA-4 antagonist antibody for T cells, it can trigger a direct effect on melanoma tumor by inducing activation of cytolytic effector cells; ii) the possibility that different CTLA-4 levels on melanoma tissues could contribute to the heterogeneous patterns of clinical response that characterize the CTLA-4 immunotherapy in metastatic melanoma patients.

## Abbreviations

ADCC: Antibody-dependent cellular cytotoxicity; APC: Antigen presenting cells; CTLA-4: Cytotoxic T Lymphocyte-Associated antigen 4; ELISA: Enzyme-linked immunosorbent assay; FFPE: Formalin-fixed, paraffin-embedded; IHC: Immunohistochemistry; IRS: Immune Reactive Score; mAbs: Monoclonal antibodies; MDA: Melanocyte differentiation antigens; MFI: Mean fluorescence intensity; MRFI: Mean ratio of relative fluorescence intensity; NCA: Neural crest antigens; PBMC: Peripheral blood mononuclear cells; SCA: Stem cell-related antigens; sCTLA-4: Soluble CTLA-4; SN: Supernatant; TMA: Tissue microarray; TNF-α: Tumor Necrosis Factor-α; Tregs: Regulatory T cells.

## Competing interests

PQ participated to Advisory Board from Bristol Myers Squibb, Merck Sharp and Dohme, Roche-Genentech, Glaxo Smith Kline. She received honoraria from Bristol Myers Squibb and Roche-Genentech. The other authors declare that they have no competing interests.

## Authors’ contributions

MPP and AP conceived the study, participated in its design and coordination and drafted the manuscript. PP, AM, NF, FT and LJC carried out molecular studies. SL, SB carried out cellular studies. MPP, SL, AP, MCM were involved in performing *in vivo* studies. SS, SB and SM carried out the immunoassays. VF performed the statistical analysis. GP, PC and GF carried out cell line generation from patients’ biopsies. PQ coordinated melanoma patients. All authors read and approved the final manuscript.

## Supplementary Material

Additional file 1 **Methods: Melanoma cell line generation and characterization (flow cytometry and transcript CTLA-4 analysis); immunohistochemistry of melanoma tissues and *in vivo *experiments.** Click here for file

Additional file 2 **Primers used in the quantitative RT-PCR (qRT-PCR) analysis of formalin-fixed, paraffin-embedded melanoma tissues and cell lines.** List of primers and PCR conditions. Click here for file

Additional file 3 **CTLA-4 expression in cutaneous melanoma tissues as detected by qRT-PCR.** Expression of CTLA-4 transcript in melanoma tissue, METR and FO-1 cell lines. Click here for file

Additional file 4 **Legend to Figure of Additional file 3.** CTLA-4 expression in cutaneous melanoma tissues as detected by qRT-PCR. Click here for file
