# Supplementary material for: The engagement of CTLA-4 on primary melanoma cell lines induces antibody-dependent cellular cytotoxicity and TNF-α production
Source: J Transl Med. 2013 May 1;11:108. doi: 10.1186/1479-5876-11-108 (PMC3663700; doi:10.1186/1479-5876-11-108)
Supplement: Additional file 1 — Methods: Melanoma cell line generation and characterization (flow cytometry and transcript CTLA-4 analysis); immunohistochemistry of melanoma tissues and in vivo experiments. [file 1479-5876-11-108-S1.doc]

**Additional file 1**

**Methods**

*Antibodies and flow cytometry*

Expression of surface CTLA-4 and CD56(NCAM) was analyzed by direct immunofluorescence using a Phycoerythrin (PE)-conjugated goat anti-CTLA-4 polyclonal serum (R&D Systems, Minneapolis, MN, USA) and a PE-conjugated anti-CD56 mAb (NCAM, N901 clone, from Beckman-Coulter, Miami, FL, USA). Expression of cytoplasmic CTLA-4 was analyzed by indirect immunofluorescence using the unconjugated anti-CTLA-4 murine mAb (14D3 clone, from eBioscience, San Diego, CA, USA).

Surface and intracellular CTLA-4 staining of PBMC was performed with the anti-CTLA-4 antibody preparations containing 1mg/ml purified human γ-globulin (human therapy grade, Biotest SRL, Milan, Italy) to prevent non specific binding to FcγR.

Flow cytometric analysis was performed with a FACSCalibur cytometer (Becton Dickinson, San Jose, CA, USA) using CellQuest software (Becton Dickinson). Ten thousand gated events were acquired for each cell population and antibody condition.

*Cell line culture conditions*

All melanoma cell lines were maintained in monolayer culture with complete medium consisting of RPMI1640 (Biochrom KG, Berlin, Germany) supplemented with 10% heat-inactivated fetal bovine serum (FBS; Biochrom KG), 1% antibiotic mixture (5mg/ml penicillin and 5mg/ml streptomycin stock solution), 2mM L-glutamine (Biochrom KG), at 37°C in a humidified 5% CO_2_ atmosphere and subcultured every 3 to 7d. The confluent cells were harvested after treatment with trypsin/EDTA (Biochrom KG) solution and diluted with medium for further assays. C1R-neo cell line was grown in complete RPMI1640 culture medium.

For culturing tumor cells from murine xenografts, freshly harvested tumors were placed into a Petri dish, finely scissor-minced and digested with 1mg/ml type I collagenase (Sigma-Aldrich, Milan, Italy) in serum-free RPMI1640 medium for 2hs at 37°C. After digestion, isolated cells were washed and seeded in 6-well flat bottom culture plates (Corning Incorporated, Corning, NJ, USA) containing culture medium and maintained in a humidified atmosphere of 5% CO_2_ in air at 37°C. These tumor cell cultures exhibited an *in vitro* morphology, growth kinetics and expression markers similar to that of the original cell line (data not shown).

*Characterization of melanocyte differentiation antigens*

The expression of melanocyte differentiation antigens (MDA) was analyzed by indirect immunofluorescence staining and flow cytometry using the following mAbs: anti-Melan-A/MART-1 (Alo3 clone) and anti-GP100 (HMB45 clone, both from Ventana Medical System, S.A. Strasbourg, France), followed by the addition of a FITC-conjugated, isotype-specific, goat anti-mouse IgG secondary antibody (D.B.A. Italia s.r.l., Segrate, Milan, Italy). Negative controls included unlabeled isotype-matched irrelevant mAbs.

*Total RNA and cDNA preparations*

Total RNA from melanoma cell lines was extracted by Qiazol (Invitrogen Life Technologies, Milan, Italy) and miRNeasy Mini Kit (Qiagen, Milan, Italy) according to manufacturer’s instructions. High Pure RNA paraffin Kit (Roche Diagnostic GmbH, Germany) was used to extract RNA from 10μm sections of FFPE melanoma tissue, as indicated in manufacturer’s instructions. Each section was processed separately and RNAs were pooled together, at the end of extraction. cDNAs were synthetized from 1μg of total RNA using SuperScript II (Invitrogen) and pentadecamer priming, in 20μl of final volume.

*Analysis of CTLA-4 transcripts by RT-PCR and qRT-PCR*

Analysis of CTLA-4 transcript variants by RT-PCR was carried out using primers specific for both the CTLA-4TM (variant 1, genBank accession numbers: NM_005214) and CTLA-4delTM (sCTLA-4, variant 2, genBank accession numbers: NM_001037631).

Amplification of CTLA-4 transcripts was performed in a reaction mix, final volume of 30μl, containing 150ng of cDNA, 0.1 mmol/L dNTPs, 2U AmpliTaq Gold (Applied Biosystems, Roche, Milan, Italy), its specific buffer with 1.5 mmol/L MgCl2 and 0.25 μmol/L of each primer, which amplify both variants (Ex2 F: 5’ ACT ATC CAA GGA CTG AGG GCC AT 3’ and Ex4 R: : 5’CAT TCT GGC TCT GTT GGG GGC3; from TIB MOLBIOL S.r.l., Genoa, Italy). PCR was performed at 68°C for 40 cycles (25” extension at 72°C). Twenty-five μl of PCR products were separated by standard electrophoresis on a 2.5% agarose gel. The remaining 5μl were cleaned up with Exo-Sap (ExoSAP-IT™, GE Healthcare Bio-Sciences, Milan, Italy), diluited 1:10 and 3μl of this dilution were used as template to amplify GAPDH (28 cycles at 54,5°C, 25” extension) in a 20μl of PCR reaction containing 0.25 μmol/L of primers (Additional file 2).

Bands corresponding to molecular weight of 290bp and 180bp were obtained for CTLA-4TM and CTLA-4delTM transcripts, respectively. Densitometric analyses of the bands were carried out with the ImageJ software. Each band of CTLA4 transcripts (TM and delTM) was normalized to the corresponding GAPDH. RT-PCR experiments were repeated three times with similar results.

Quantitative Real-Time PCR (qRT-PCR) assays were performed in StepOne Plus machine (Applied Biosystems, Roche, Milan, Italy), in 20μl reaction containing dilution of cDNA samples, Express SYBR® GreenER qPCR mix and specific primers (Additional file 2). At the end of the 40 cycles of PCR at 58°C, the melt curve was made by increasing the temperature from 60°C to 95°C (ramp rate of 0.3°C). Expression of CTLA4 transcripts, normalized on GAPDH and RP2 housekeeping gene expression (by BestKeeper software, http://www.wzw.tum.de/gene-quantification/bestkeeper.html) was calculated by Q gene software (http://biotechniques.com/softlib/qgene.html) as mean of normalized expression (±S.E.M.).

qRT-PCR assay was performed twice on two different preparations of cDNAs and all samples were analysed in triplicate. Specificity of PCR products was previously confirmed by direct sequencing analysis, in both directions, using an ABI-PRISM 3130 (Applied Biosystems, Foster City, CA, USA).

*Iimmunohistochemistry*

Melanoma cell lines and 4-μm thick FFPE tissue sections were subjected to antigen retrieval by heating with citrate buffer (pH 7) at 90°C for 30min followed by staining with the murine 14D3 or the human Ipilimumab anti-CTLA-4 mAbs.

In the case of 14D3, 10µg/ml (final concentration) of the mAb were added using the automatic immunostaininer Benchmark XT (Ventana Medical Systems, SA Strasbourg, France) and incubated for 40min at 37°C. Reactivity detection was performed by addition of the polymeric system Ultraview Universal Alkaline Phosphatase (AP) Red Detection Kit (Ventana Medical System) as this system allows to distinguish between red positive cells and brown melanin pigment.

In addition to conventional whole sections, melanoma tissue microarrays (TMA; Biomax, Inc., Rockville, MD) were also stained with 14D3 mAb using the same methodology.

In the case of Ipilimumab, 50µg/ml (final concentration) of the mAb were added and incubated for 1hr at room temperature. As the automatic Ventana system does not allow the use of secondary anti-human Abs, Ipilimumab reactivity was detected by adding manually an anti-human horseradish peroxidase (HRP)-conjugated secondary antibody (D.B.A. Italia s.r.l.) and DAB (3.3’-diaminobenzidine) as chromogen that gives brown staining in positive cells.

In both cases, the sections were counter-stained with Mayer’s hematoxylin and then cover-slipped. Positive controls for melanoma cells and tissues were obtained by staining with the anti-S100 melanoma marker antibody (4c4.9 clone, Ventana Medical Systems), followed by addition of the polymeric system Ultraview Universal AP Red Detection Kit.

Isotype controls were obtained by staining with a murine anti-CD20 IgG2a (L26 clone, Ventana Medical Systems) and a human anti-CD20 IgG1 (Rituximab) as isotype-matched irrelevant mAbs for both 14D3 and 4c4.9 and for Ipilimumab, respectively.

*Leukocyte cell separation, antibody-dependent cellular cytotoxicity (ADCC) and TNF-α production assays*

PBMC were obtained after Ficoll-Hypaque density centrifugation of blood samples derived from healthy volunteers as previously described (8). NK cells were isolated from PBMC using the negative selection Rosettesep NK isolation kit (Stemcell Technologies, Vancouver, Canada) according to manufacturer’s instruction. Ex-vivo isolated NK cell populations were 70-95% CD16^+^CD56^+^ and 100% CD3^-^ (n=8). In some experiments, ex-vivo NK cells were stimulated with PHA (1μg/ml) and cultured with IL-2 (10ng/ml) for 3wks to obtain highly enriched NK cell bulk populations (100% CD3^-^CD16^+^CD56^+^ n=8). γδT cells were obtained from PBMC using positive selection with Easysep do-it-yourself separation kit (Stemcell Technologies) using an anti-Vδ2 specific antibody as described (35). Purity of Vδ2^+^T cells ranged from 90-98%. Vδ2^+^T cells were then expanded with IL-2 (5ng/ml) for 3wks to obtain Vδ2^+^ bulk populations (100% Vδ2^+^ 15-80% CD16^+^CD56^+^). PBMC, ex-vivo and bulk NK, as well as bulk Vδ2^+^ populations were used as effector cells in ADCC assay. This assay was performed by adding Ipilumumab or Rituximab antibodies (20-2.0-0.2μg/ml) to a 4h standard cytolytic assay. Tumor target cells were labelled with sodium ^51^Chromate, seeded in 96-V-bottomed microplates with effector cells (E)/target (T) at different E:T ratios in 200μl of volume and specific lysis was calculated as described (36). In some experiments, an anti-FcγRIIIA mAb (anti-CD16 mAb:KD1 clone, 5μg/ml) or an anti-NCAM mAb (anti-CD56 mAb:TA181H12 clone 5μg/ml, both produced in our laboratory) was added at the onset of the cytolytic assay to block the interaction of Ipilimumab with FcγRIIIA. PHA-stimulated PBMC were also used as target cells in ADCC assays with ex-vivo isolated NK cells.

Production of TNF-α was determined by ELISA from SN of NK-melanoma cells cultured at 1:1 ratio alone or with Ipilimumab or Rituximab at 37°C for 24h. Two μg/ml of Ipilimumab was the optimal antibody amount in inducing TNF-α production as determined in preliminary experiments. The amount of TNF-α in each SN was calculated from a standard curve determined with recombinant TNF-α.

*In vivo tumorigenicity assay*

For tumorigenicity assay, exponentially growing melanoma cells at different cellular concentrations (1-10x10^6^), were resuspended in 200µl of PBS and injected subcutaneously (s.c.) into NOD/SCID mice (6 injections per each experimental condition). Tumor growth was monitored twice a week until tumor appearance (within 10-15 days), then nodule diameters were measured *in vivo* by external caliper bi-weekly for up to 30 days. Tumor growth was evaluated with the formula: V (mm^3^) = (D×d2)/2, where d (mm) and D (mm) are the smallest and largest perpendicular tumor diameter, respectively. Growth rates were determined by plotting the mean tumor volume (mm^3^) *versus* time.
