# Supplementary material for: The engagement of CTLA-4 on primary melanoma cell lines induces antibody-dependent cellular cytotoxicity and TNF-α production
Source: J Transl Med. 2013 May 1;11:108. doi: 10.1186/1479-5876-11-108 (PMC3663700; doi:10.1186/1479-5876-11-108)
Supplement: Additional file 2 — Primers used in the quantitative RT-PCR (qRT-PCR) analysis of formalin-fixed, paraffin-embedded melanoma tissues and cell lines. List of primers and PCR conditions. [file 1479-5876-11-108-S2.doc]

| **Additional file 2**  **Table. Primers used in the quantitative RT-PCR (qRT-PCR) of formalin-fixed, paraffin-embedded melanoma tissues and cell lines.** | | | | |
| --- | --- | --- | --- | --- |
| **qRT-PCR** | | | | |
| **PCR products** | Primers | Primer concentration (nM) | Melt Temp.  (+/-0.5°C) | Amplicon size (bp) |
| **CTLA4 (TM-delTM)** | Ex1 Forward: 5’ TCT TCA TCC CTG TCT TCT 3’ | 250 | 86 | 95 |
|  | Ex2 Reverse: 5’ ATA CTC ACA CAC AAA GCT G 3’ | 250 |  |  |
|  |  |  |  |  |
| **GAPDH** | Forward: 5’ TGA ACC ATG AGA AGT ATG AC 3’ | 400 | 82 | 110 |
|  | Reverse: 5’ CAC GAT ACC AAA GTT GTC 3’ | 400 |  |  |
|  |  |  |  |  |
| **RP2** | Forward: 5’ CGC TTA CTG TCT TCC TGT 3’ | 200 | 83 | 129 |
|  | Reverse: 5’ GGT TGG GGT CAT AGT AGA 3’ | 200 |  |  |
