# Supplementary material for: The engagement of CTLA-4 on primary melanoma cell lines induces antibody-dependent cellular cytotoxicity and TNF-α production
Source: J Transl Med. 2013 May 1;11:108. doi: 10.1186/1479-5876-11-108 (PMC3663700; doi:10.1186/1479-5876-11-108)
Supplement: Additional file 3 — CTLA-4 expression in cutaneous melanoma tissues as detected by qRT-PCR. Expression of CTLA-4 transcript in melanoma tissue, METR and FO-1 cell lines. [file 1479-5876-11-108-S3.ppt]

## Slide 1
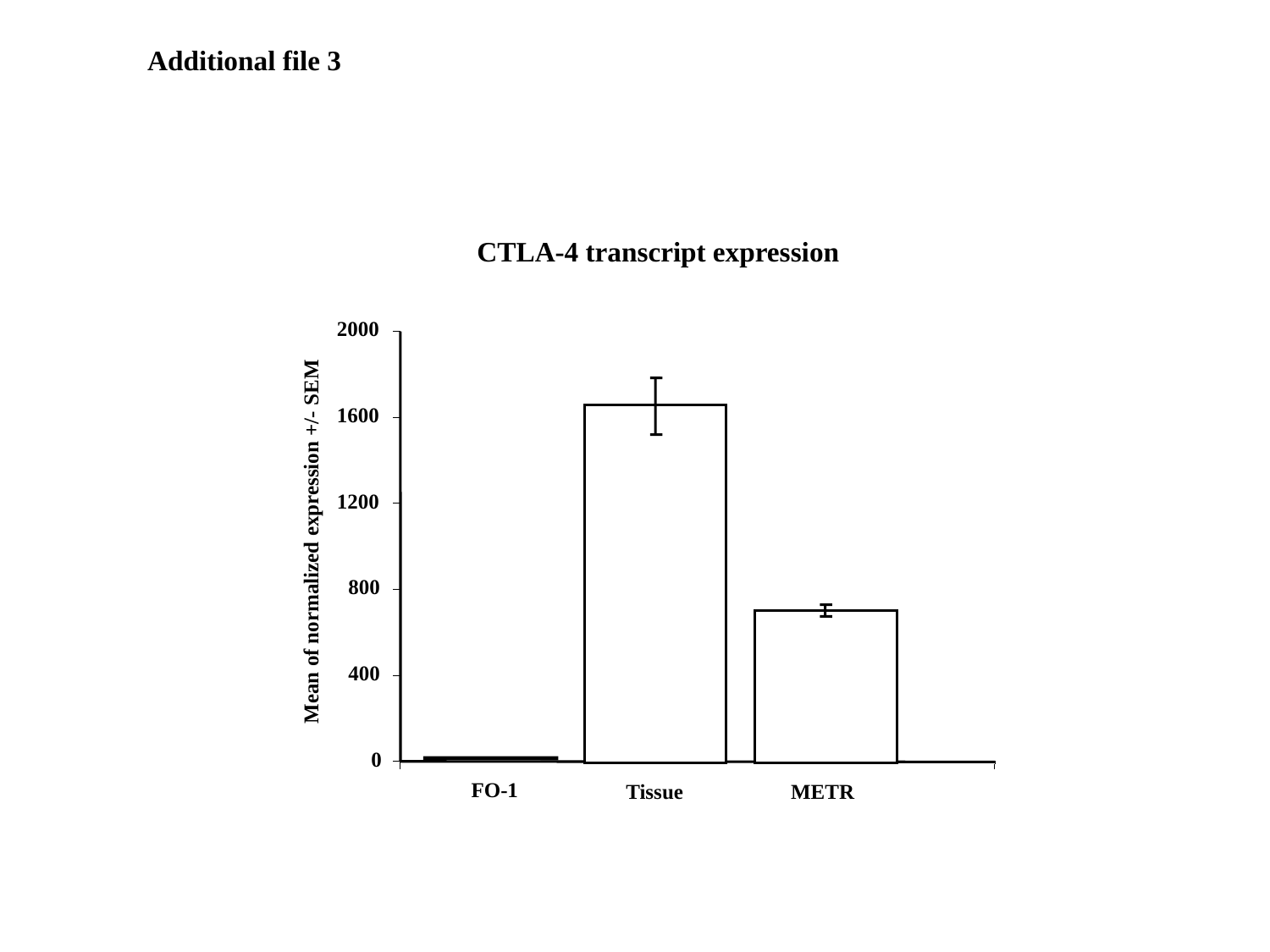

Additional file 3
CTLA-4 transcript expression
2000
1600
1200
Mean of normalized expression +/- SEM
800
400
0
FO-1
Tissue
METR
