# Supplementary material for: The engagement of CTLA-4 on primary melanoma cell lines induces antibody-dependent cellular cytotoxicity and TNF-α production
Source: J Transl Med. 2013 May 1;11:108. doi: 10.1186/1479-5876-11-108 (PMC3663700; doi:10.1186/1479-5876-11-108)
Supplement: Additional file 4 — Legend to Figure of Additional file 3. CTLA-4 expression in cutaneous melanoma tissues as detected by qRT-PCR. [file 1479-5876-11-108-S4.docx]

**Additional file 4**

**Legend to Figure of Additional file 3**

CTLA-4 expression in cutaneous melanoma tissues as detected by qRT-PCR. Quantitation of CTLA-4 transcript expression by qRT-PCR was performed on formalin-fixed, paraffin-embedded cutaneous melanoma tissues. Results from a representative tissue sample are shown, together with results from FO-1 and METR melanoma cell lines. QRT-PCR was performed with specific primers as described in Additional file 2. Results are expressed as mean of normalized expression (MNE±S.E.M) of duplicate experiments and plotted versus FO-1. An arbitrarly value of 1, corresponding to a MNE of 3.45E^-6^(±1.93E^-8^), was attributed to FO-1.
